# Plasma lipid alterations in thrombosis and antiphospholipid syndrome—A differentiating lipid plasma composite score

**DOI:** 10.1016/j.jlr.2026.101115

**Published:** 2026-07-31

**Authors:** Susanne Heimerl, Christina Hart, Marcus Höring, Ralph Burkhardt, Matthias Höpting, Gerhard Liebisch

**Affiliations:** 1 Institute of Clinical Chemistry and Laboratory Medicine, University Hospital Regensburg, Regensburg, Germany; 2 Department of Hematology and Oncology, Internal Medicine III, University Hospital Regensburg, Regensburg, Germany

**Keywords:** lipidomics, quantitative mass spectrometry, alkyl-phosphatidylcholine, lipid species biomarker, glycerophospholipids, sphingolipids

## Abstract

Antiphospholipid syndrome (APS) is an autoimmune thrombophilia diagnosed by established clinical and serological criteria, distinguishing it from other causes of venous thromboembolism (VTE). This study investigated whether VTE is associated with plasma lipidome changes and whether an APS-specific lipid signature can be identified. We performed quantitative mass spectrometry-based lipidomics on plasma from healthy controls (n = 16), patients having VTE without APS (n = 16) and triple-positive APS patients (n = 17), profiling 224 lipid species across all major classes. Ceramide species (Cer 18:1;O2/16:0,/18:0,/20:0 and/26:0) and the Cer 18:0/Cer 24:0 ratio were elevated in both VTE groups relative to healthy controls, consistent with a shared thrombotic state. To identify APS-specific changes, LASSO logistic regression defined a minimal discriminating lipid panel. PE 40:5 (elevated 1.69-fold in APS), SM 43:2;O2 and PC O-34:2 (both reduced by 21%–24%) were combined into a parameter-free composite score that discriminated APS from VTE without APS with an AUC of 0.857. PE 40:5 has previously been associated with cardiovascular inflammatory activation, whilst reduction of PC O-34:2 may reflect increased conversion to platelet-activating factor and/or plasmalogen consumption under the pro-inflammatory conditions of APS. The co-occurrence of elevated ceramides and decreased SM 43:2;O2 in APS raises the hypothesis of enhanced acid sphingomyelinase activity driving sphingomyelin-to-ceramide conversion. Prospective validation in larger cohorts is warranted to assess its utility as a complement to serological APS diagnostics. In conclusion, despite the limited sample size, plasma lipidomics identifies a three-lipid composite score that discriminates APS from VTE without APS.

Venous thromboembolism (VTE), including deep vein thrombosis (DVT) and pulmonary embolism is a common and significant preventable cause of morbidity and mortality on a global scale (reviewed in (1)). In Western countries, the incidence of VTE is reported to be approximately 150 per 100,000, with a death rate of approximately 26 per 100,000 (reviewed in (2)). Numerous factors play a role in the development of thrombosis. In addition to conditions like surgery, malignant diseases, pregnancy, increasing age, and obesity, congenital thrombophilic risk factors such as the Factor V Leiden mutation or a deficiency of coagulation inhibitors such as protein S and C or antithrombin can also promote the development of thrombosis (reviewed in (1)). A specific underlying disease associated with a high risk of thromboembolic events is antiphospholipid syndrome (APS), an immune-mediated thrombo-inflammatory disorder. APS is characterized by venous and/or arterial thromboembolic events and/or obstetric complications in the presence of antiphospholipid antibodies (aPL) comprising anticardiolipin antibodies (aCL), anti-β2-glycoprotein I antibodies (aβ2GPI) and the functionally measured lupus anticoagulant (LA) (reviewed in (3, 4)).

In addition to well-established thrombophilia risk factors, such as inhibitor deficiency or antiphospholipid antibodies, there is also evidence that certain (minor) plasma lipids including glucosylceramide, lysosulfatide, sphingosine, lysophospholipids and ethanolamides may influence the risk of thrombosis (reviewed in (5)). Despite the fact that the main carrier of lipids in plasma are lipoproteins, studies investigating a potential link between thrombosis and classic lipoproteins have only been partially successful. The Longitudinal Investigation of Thromboembolism Etiology (LITE) prospectively assessed VTE risk in relation to baseline high-density lipoprotein (HDL) cholesterol in 19,049 participants and did not demonstrate an apparent trend in VTE incidence across HDL cholesterol quartiles (6). Similar results were found in the MEGA-study, which included 2,234 patients with initial thrombosis and 2,873 controls. Although the study reported a moderate association between thrombosis risk and decreased levels of apolipoproteins B and A1, no such association was observed for total cholesterol, LDL cholesterol, HDL cholesterol, and triglycerides (7). These findings were recently confirmed in a Mendelian randomization study, which found no evidence of a causal relationship between LDL cholesterol, HDL cholesterol or triglycerides and VTE. Furthermore, the study identified no significant causal effects of VTE on these lipids (8).

Although no significant association between VTE and classic plasma lipids and lipoproteins has been demonstrated to date, emerging lipidomic approaches allow the detection of changes in specific lipid species in VTE. A recent large-scale two-sample Mendelian randomization analysis has indicated an association between fatty acid unsaturation traits and VTE, DVT, and pulmonary embolism. Furthermore, the authors identified a protective effect of medium sized LDL on VTE, and were able to distinguish specific phosphatidylcholine species as exhibiting positive and negative associations with VTE and pulmonary embolism (9).

However, only limited data exist on specific alterations of the plasma lipidome in VTE, and the plasma lipidome of patients with APS, a specific thrombophilic condition, has not yet been investigated. Therefore, using quantitative mass spectrometry-based lipidomics, we here aimed to investigate alterations in the plasma lipidome of VTE patients with and without APS.

## Materials and Methods

### Patients’ characteristics and ethical approval

Plasma samples were obtained from patients and healthy volunteers, all of whom had discontinued antiplatelet therapy for at least 10 days prior to sample collection. Patients undergoing statin therapy were excluded from the study. Patients with APS were required to have experienced at least one venous or arterial thromboembolic event diagnosed at least 12 weeks before study inclusion, as well as persistent moderate to high titer aCL and aβ2GPI of IgG or IgM isotypes and the detection of a functionally measured lupus anticoagulant (LA), in accordance with established classification and laboratory criteria for APS (10, 11). According to the modified Sapporo criteria, all patients were classified as triple-positive (11), and 85% of all patients fulfilled the criteria for triple-positivity when using the American College of Rheumatology/European Alliance of Associations for Rheumatology criteria, published in 2023 (12). In 90% of the patients with APS, there was no underlying disease and the APS occurred as a primary condition. Patients affected by venous thromboembolism with no clinical or laboratory evidence for APS were additionally included in the study. The intake of direct oral anticoagulants (DOACs) had to be paused for at least 48 h before blood sampling; otherwise, the plasma sample was pretreated with DOAC Stop (Haematex) to remove the interference of DOACs on clotting assays (13).

The study was approved by the local Ethics Committee (Universitätsklinikum Regensburg, Ethikkommission der medizinischen Fakultät, proposal 20-1836-101). All subjects had given their informed consent to participate in the study. All research was performed in accordance with the relevant guidelines and regulations, including the Declaration of Helsinki.

### Quantification of aPL and LA

aCL and aβ2GPI of IgG or IgM were quantified in serum on a BIO-FLASH® Chemiluminescent Analyzer (Inova Diagnostics) using aCL IgM, aCL IgG, β2GPI IgM and β2GPI IgG QUANTA Flash® Reagents (Inova Diagnostics) according to the manufacturer's instructions.

The presence of LA was detected by two different assay principles, diluted Russell viper venom time (DRVVT) (LA1 Screening Reagent/LA2 Confirmation Reagent, Siemens Healthineers) and aPTT (HemosIL® APTT-SP/HemosIL® SynthAFax, Instrumentation Laboratory, Werfen). All clotting tests were analyzed on BCS®-XP (Siemens Healthineers).

### Blood count

Blood count including platelet count was analyzed on a hematology analyzer (XN-10/XN-20, Sysmex Corporation) according to the manufacturer’s instructions.

### Lipid mass spectrometry

Lipid extraction was performed according to the method of Bligh and Dyer (14) in the presence of not naturally occurring lipid species as internal standards. A volume of 10 μl EDTA plasma was subjected to extraction.

The analysis of lipids was performed by direct flow injection analysis (FIA) using a triple quadrupole mass spectrometer (FIA-QQQ) and a hybrid quadrupole-Orbitrap mass spectrometer (FIA-FTMS).

FIA-QQQ was performed using the analytical setup and strategy described previously (15). The following lipid classes were quantified by FIA-QQQ: A fragment ion of *m/z* 184 was used for lysophosphatidylcholines (LPC) (16) phosphatidylethanolamine (PE) and lysophosphatidylethanolamine (LPE) using neutral loss of 141 Da, and phosphatidylinositol (PI) using a precursor ion scan of 277 Da, as well as PE-based plasmalogens (PE P) (17). Sphingosine-based (18:1;O2) ceramides (Cer) and hexosylceramides (HexCer) were analyzed using a fragment ion of *m/z* 264 (18).

The Fourier Transform Mass Spectrometry (FIA-FTMS) setup is described in detail in Höring *et al.* (19). The following lipid classes were quantified by FIA- FTMS: Triglycerides (TG), diglycerides (DG), cholesteryl ester (CE), phosphatidylcholine (PC) and sphingomyelin (SM) as well as free cholesterol (FC) (20).

Lipid species were annotated according to the latest proposal for shorthand notation of lipid structures derived from mass spectrometry (21). For QQQ, glycerophospholipid species annotation was based on the assumption of even-numbered carbon chains only.

Details of the lipidomics analysis are described in the attached Lipidomics Reporting Checklist (22) for FIA-FTMS (supplemental PDF 1) and FIA-QQQ (supplemental PDF 2). Furthermore, we provided details on the internal standards that were added in supplemental Table S1.

To identify the molecular species of lipids, the product ion spectra of the regulated lipid species were analyzed using the FIA-FTMS setup (19) with higher-energy collisional dissociation (HCD)-MS/MS. Briefly, formiate adducts [M+HCOO]^-^ of PC O were analyzed at 30% HCD; deprotonated ions [M-H]^-^ of PE and PI at 30% and 35% HCD, respectively. [M+H]^+^ ions of SM were fragmented at 20% HCD for lipid class specific production ion of *m/z* 184 and at 30% for long chain base fragments.

### Statistical analysis

Data are expressed as mean ± SD in bar graphs or as median ± min/max in box plots. Statistical analysis was performed using IBM SPSS statistics 22 software. Groups were compared using the Kruskal-Wallis test followed by Dunn’s nonparametric comparison for post*-*hoc testing. The *P*-values were corrected for multiple testing using the Bonferroni procedure. A *P*-value <0.05 was considered statistically significant.

Volcano plots were generated with RStudio (version 2023.6.2) using R (version 4.1.2). A Student’s *t* test was performed for the calculation of *P*-values. These *P*-values are uncorrected for multiple comparisons and were used for exploratory visualization only.

### Lipid signature identification

Lipidomics data (nmol/ml) were log2-transformed prior to analysis. Univariate between-group comparisons were performed using two-tailed Student's t-tests; *P*-values were adjusted for multiple testing using the Benjamini–Hochberg false discovery rate (BH-FDR) procedure. Lipids with FDR-adjusted *P* < 0.05 and an absolute fold change ≥1.25 were considered statistically significant. (results reported in supplemental Table S2).

To identify a minimal lipid panel discriminating APS from VTE without APS, LASSO (Least Absolute Shrinkage and Selection Operator) logistic regression was applied to all 224 log2-transformed species using the glmnet package (v4.1-10). The regularization parameter λ was determined by 5-fold cross-validation minimizing misclassification error; the most parsimonious model within one standard error of the minimum (λ.1se) was selected to favor sparsity and reduce overfitting. Discriminative performance was assessed by ROC analysis using the pROC package (v1.19.0.1); the area under the curve (AUC) with 95% confidence interval (DeLong method) is reported. All analyses were performed in R (v4.5.2, R Foundation for Statistical Computing, Vienna, Austria).

## Results

### Comparison of lipid alterations in thrombosis and healthy blood donors

**Fig. 1:**
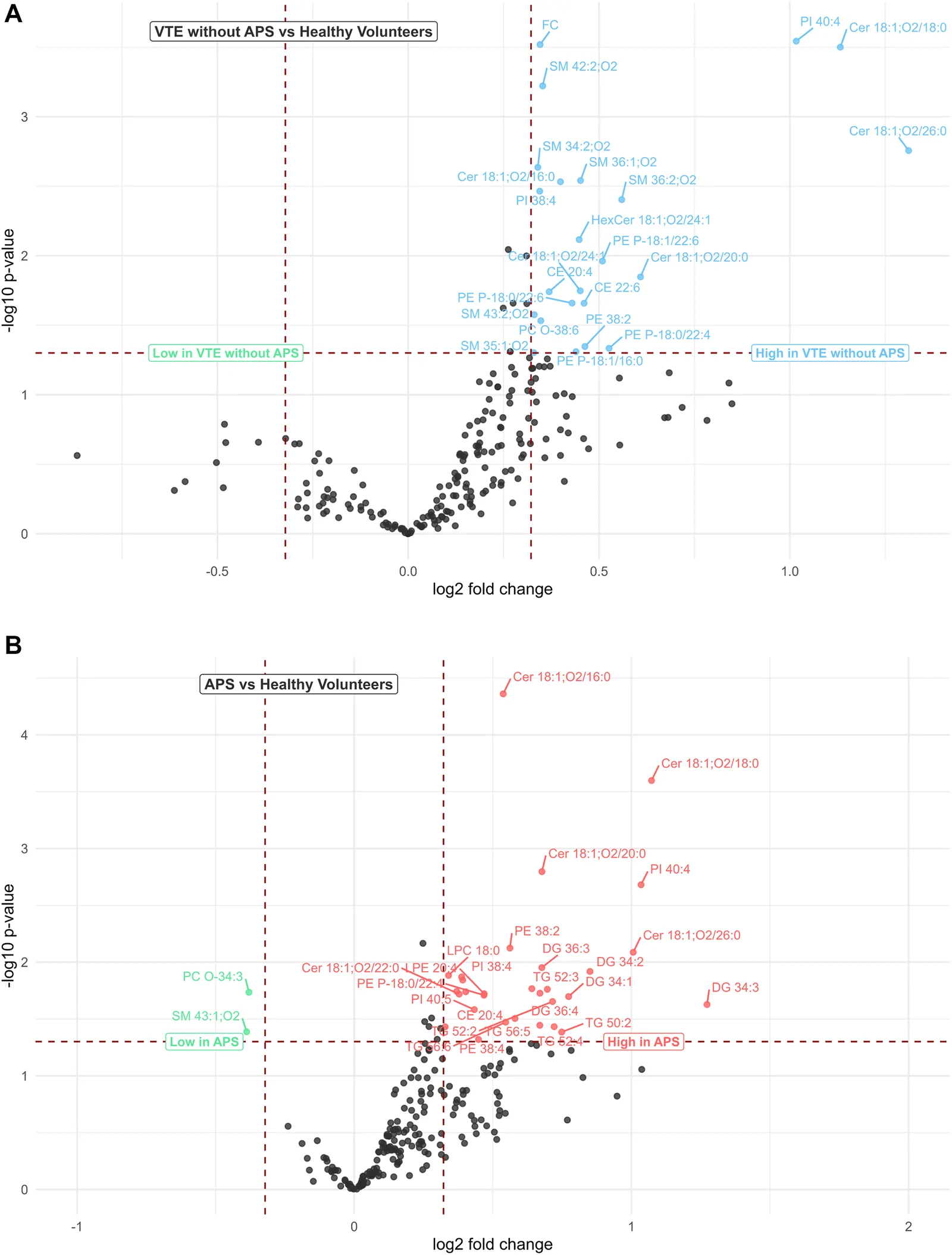
Lipid alterations in thrombosis patients compared to healthy blood donors. Plasma of healthy volunteers (n = 16), VTE patients without APS (n = 16) and APS patients (n = 17) was subjected to mass-spectrometry based quantitative lipidomics (data evaluation included PC, PC O, SM, DG, TG, CE FC analyzed by FIA-FTMS and LPC, LPE, Cer, HexCer, PE, PE P, PI quantified by FIA-QQQ). A: Volcano Plot comparing concentrations of all 224 measured lipid species in VTE patients without APS and healthy volunteers, and (B) APS patients and healthy volunteers, respectively. The dashed lines indicate *P* ≤ 0.05 (y-axis; Student’s *t* test, not corrected for multiple comparisons; shown for exploratory visualization) and fold changes ± 25% (x-axis).

Plasma samples were obtained from healthy volunteers (n = 16), patients with venous thromboembolism (VTE, n = 16) without antiphospholipid syndrome (APS), and patients with triple-positive antiphospholipid syndrome (APS, n = 17) (Tables 1 and 2). These samples were then subjected to quantitative lipidomics analysis. In an untargeted approach, we evaluated the differences of all 224 quantified lipid species and classes in VTE patients without APS and APS patients compared to healthy volunteers, respectively. This approach indicated increased concentrations of Cer 18:1;O2/18:0, Cer 18:1;O2/26:0 and phosphatidylinositol (PI) 40:4 in VTE patients without APS as compared to healthy volunteers (Fig. 1A). These lipid species were also identified as increased in APS plasma samples compared to healthy volunteers. Moreover, concentrations of Cer 18:1;O2/16:0, Cer 18:1;O2/20:0, as well as the diacylglycerol (DG) species DG 34:2 and DG 34:3 were elevated in APS. In contrast, ether-phosphatidylcholine (PC-O) 34:3 and sphingomyelin (SM) 43:1;O2 were identified as decreased in APS in this first approach (Fig. 1B).

**Table 1:** Main characteristics of the patient population and the healthy volunteers

	Patients with APS	Thrombosis patients without APS	Healthy Volunteers
Number	17	16	16
Sex, n (%)			
female	13 (76.5)	8 (50)	10 (62.5)
male	4 (23.5)	8 (50)	6 (37.5)
Age			
mean (years)	44.8	45.5	41.1
standard deviation	13.3	14.3	9.8
range (years)	23–64	27–61	27–56
BMI			
mean	30.7	28.7	23.9
standard deviation	6.9	6.9	4.5
range	21.3–43.0	21.1–41.8	19.9–35.3

**Table 2:** Patients` characteristics based on thromboembolic events, and laboratory values

	Patients with APS	Thrombosis patients without APS
Number	17	16
Clinical manifestation of hypercoagulability		
Venous thromboembolism	13	16
One event, n (%)	7 (54)	16 (100)
Two events, n (%)	4 (31)	
Three events, n (%)	2 (14)	
Arterial thromboembolism	7	
In addition to a venous thromboembolic event, n (%)	4 (57)	
Solely arterial, n (%)	3 (43)	
Laboratory values		
Thrombocytes per nl (median, range)	268 (172–362)	262 (154–417)
Anti-Cardiolipin IgG (CU), (median, range)	212 (5–1405)	b
Anti-Cardiolipin IgM (CU), (median, range)	9 (2–2085)	c
Anti-beta2-GP IgG (CU), (median, range)	604 (29–11449)	c
Anti-beta2-GP IgM (CU), (median, range)	7 (1–513)	c
Presence of lupusanticoagulant^a^, n (%)	17 (100)	0 (0)

CU, chemiluminescent unit.

a Laboratory testing was performed according to the laboratory criteria for APS (1, 2).

b Two patients had values slightly above the cut-off whereas the values in all other patients were under the cut-off (<20 CU) of the manufacturer`s instruction.

c All laboratory values were under the cut-off (<20 CU) of the manufacturer`s instruction.

### Ceramide profile is altered in VTE without and with APS

**Fig. 2:**
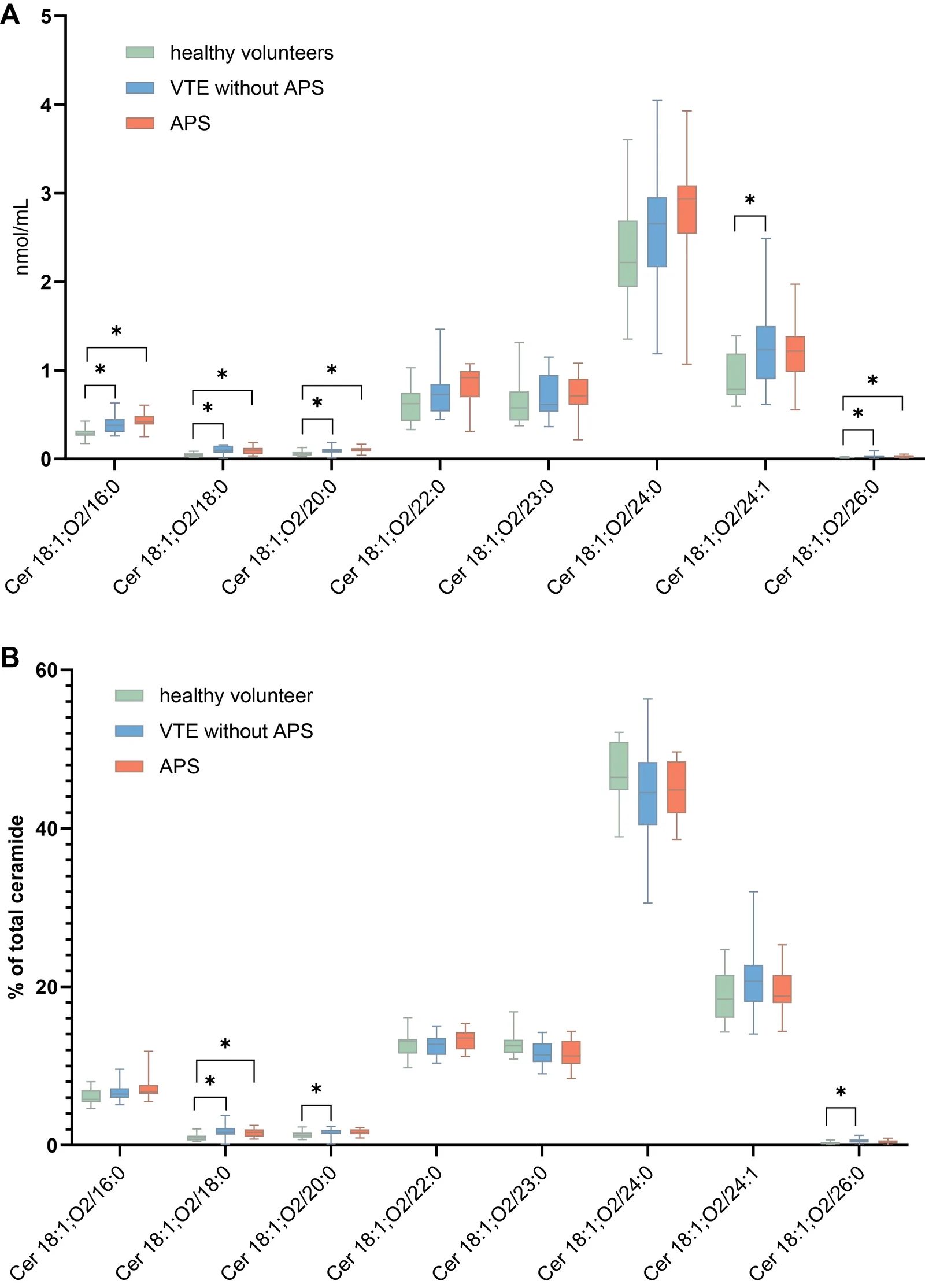
Ceramide concentration and composition. Data of ceramide concentration (analyzed by FIA-QQQ) (A) and ceramide composition (B) are expressed as median ± min/max in box plots. Groups (healthy volunteers (n = 16), VTE patients without APS (n = 16) and APS patients (n = 17)) were compared using the Kruskal-Wallis test followed by the *Dunn-Bonferroni post-hoc test*. ∗*P* < 0.05, ∗∗∗*P* < 0.001.

Since we had identified ceramides as the lipid class that had undergone the most prominent alteration, we next conducted an analysis of ceramide species. We observed significantly increased plasma levels of almost all analyzed ceramide species in patients having VTE without APS and APS patients in comparison with healthy volunteers. Cer18:1;O2/16:0, Cer18:1;O2/18:0, Cer18:1;O2/20:0, and Cer18:1;O2/26:0 were elevated in both VTE and APS patients, while Cer18:1;O2/24:1 reached significance only in VTE patients (Fig. 2A). In addition, we examined ceramide composition, defined as the proportion of individual ceramide species relative to total ceramide. Here, the proportions of the minor ceramide species Cer18:1;O2/18:0, Cer18:1;O2/20:0, and Cer18:1;O2/26:0 were also elevated in VTE and Cer18:1;O2/18:0 additionally in APS patients (Fig. 2B).

### Ceramide ratios

**Fig. 3:**
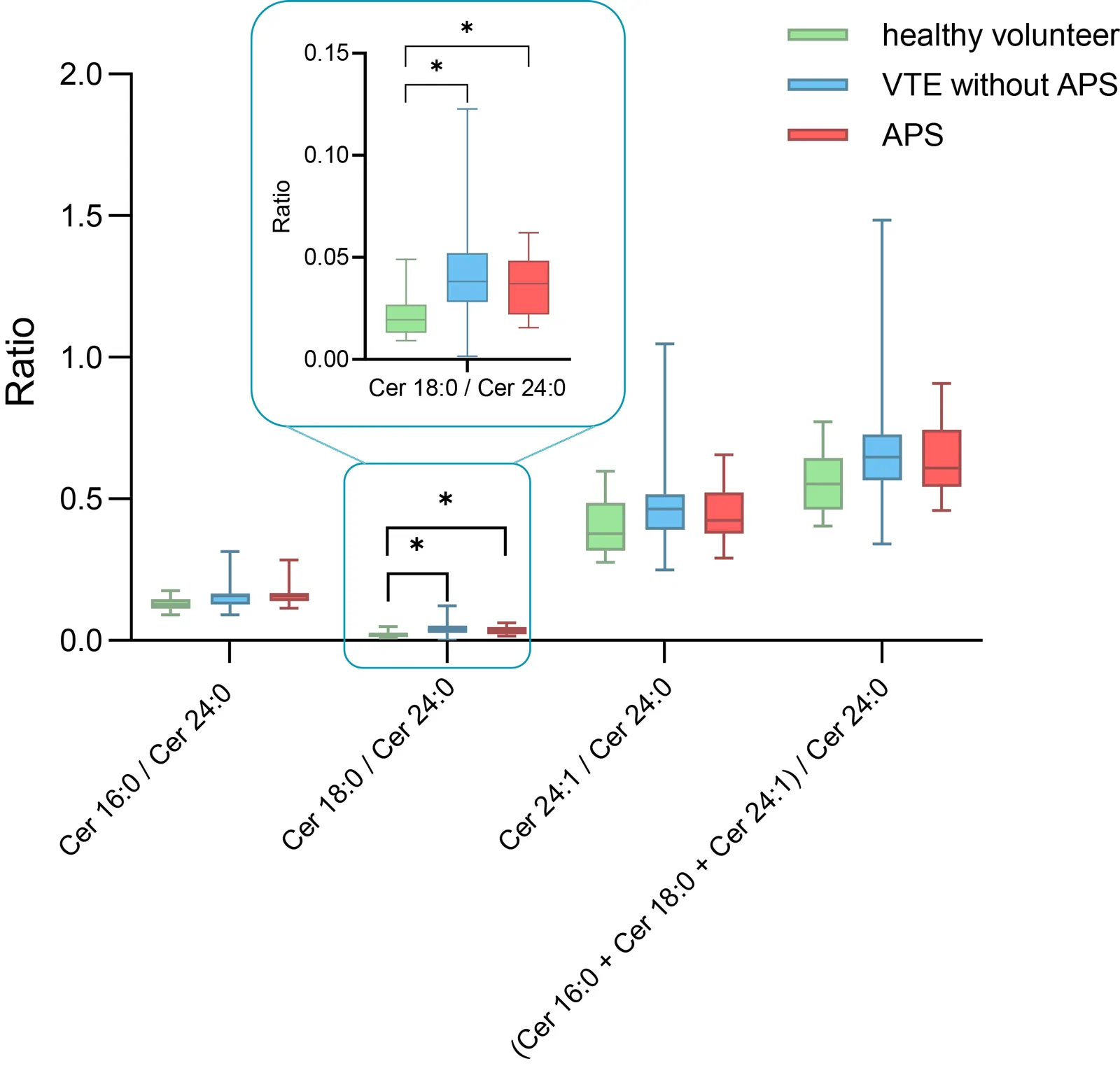
Ceramide Ratios. Data of ceramide ratios are expressed as median ± min/max in box plots. Groups (healthy volunteers (n = 16), VTE patients without APS (n = 16) and APS patients (n = 17)) were compared using the Kruskal-Wallis test followed by the *Dunn-Bonferroni post-hoc test*. ∗*P* < 0.05, ∗∗∗*P* < 0.001. For clarity, only the N-acyl chains of the ceramide species 18:1;O2 are annotated.

It has previously been demonstrated that specific ceramide ratios may serve as biomarkers for cardiovascular diseases, including coronary heart disease, stroke and type 2 diabetes mellitus. (reviewed in (23)). Since we found certain ceramide species significantly altered in VTE without APS and APS, we also calculated ratios for sphingosine-based ceramides (Cer 18:1;O2), previously implicated in vascular disease: Cer 16:0 to 24:0, Cer 18:0 to 24:0, Cer 24:1 to 24:0 and (Cer 16:0 + 18:0 + 24:1) to Cer 24:0. Compared to healthy controls (0.021), we found significantly increased Cer 18:0/Cer 24:0 ratios in VTE (*P* = 0.043) and APS (*P* = 0.036) patients (Fig. 3).

### Comparison of lipid alterations in VTE patients without and with APS

**Fig. 4:**
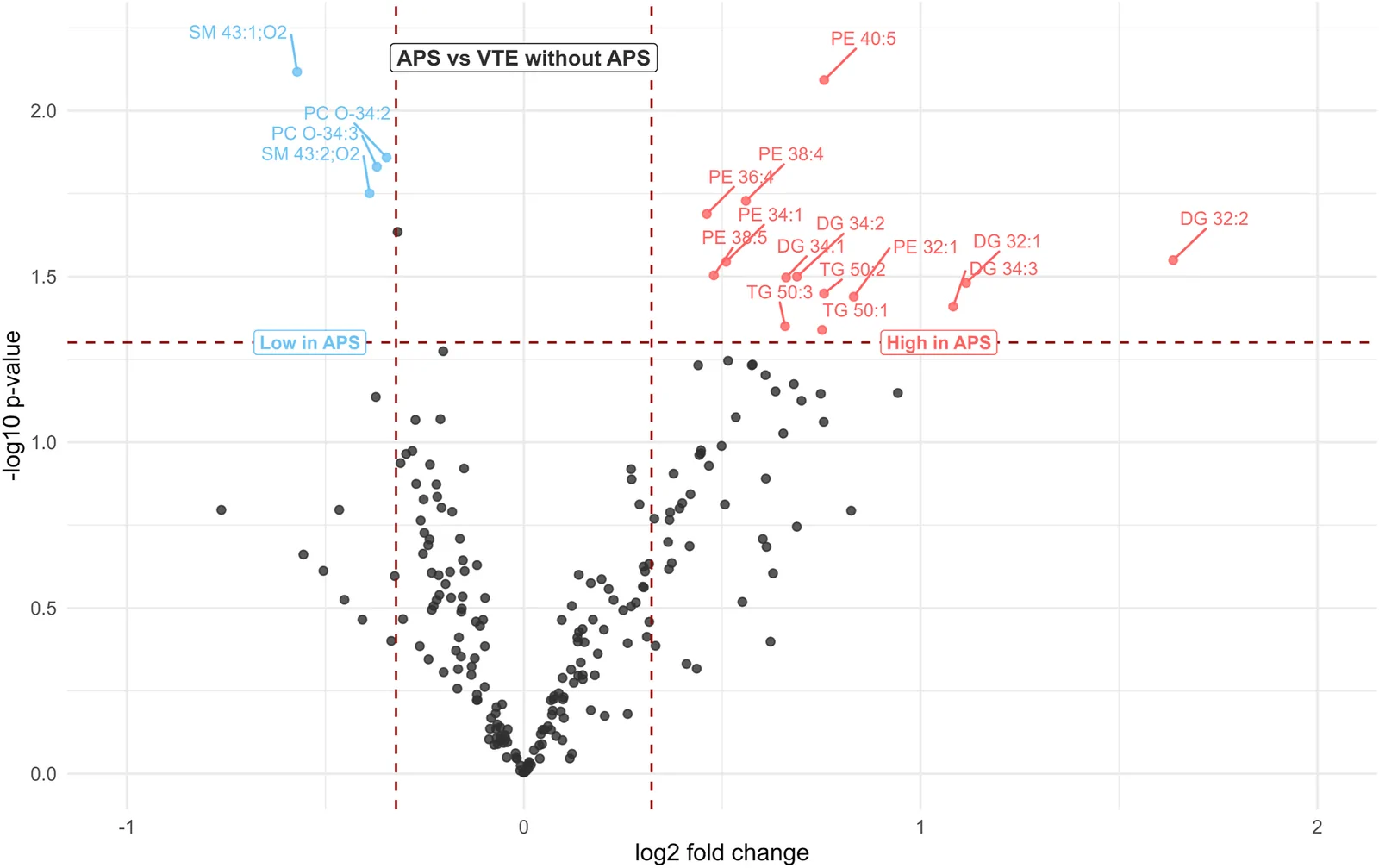
Lipid alterations in APS versus VTE. Volcano Plot comparing concentrations of all 224 measured lipid species (see Fig.1 for related analysis platform) in VTE patients without APS and APS patients. The dashed lines indicate *P* ≤ 0.05 (y-axis; Student’s *t* test, not corrected for multiple comparisons; shown for exploratory visualization) and fold changes ± 25% (x-axis).

To evaluate lipid alterations specific to APS, we compared these patients with the VTE without APS group across all 224 quantified lipid species. Plasma concentrations of several PE species (34:1, 36:4, 38:4, 38:5 and 40:5) and DG species (32:1, 32:2, 34:1 and 34:4) were elevated, whereas SM 43:1;O2, SM 43:2;O2, PC O-34:2 and PC O-34:3 were decreased in APS compared to VTE without APS (Fig. 4). These PC ether species can contain either an alkyl bond (plasmanyl-) or a vinyl ether bond (plasmenyl-, i.e., plasmalogen), which determines their biological function. To evaluate the likelihood of these bond types, we analyzed product ion spectra of three selected samples per group. While for PC O-34:2 an 18:2 acyl ion dominates (>80%; PC O-16:0/18:2), with less than 20% 18:1 of the related carboxylate product ion (PC O-16:1/18:1 or PC P-16:0/18:1; supplemental Table S3), for PC O-34:3 we found almost only an 18:2 acyl fragment ion related to PC O-16:1/18:2 or PC P-16:0/18:2. The intensities of these carboxylate ions mirrored the differences observed for the corresponding precursor ions, with lower levels in APS compared to VTE without APS. However, none of the tested species reached statistical significance after Benjamini–Hochberg FDR correction, consistent with the limited statistical power of this exploratory cohort.

**Fig. 5:**
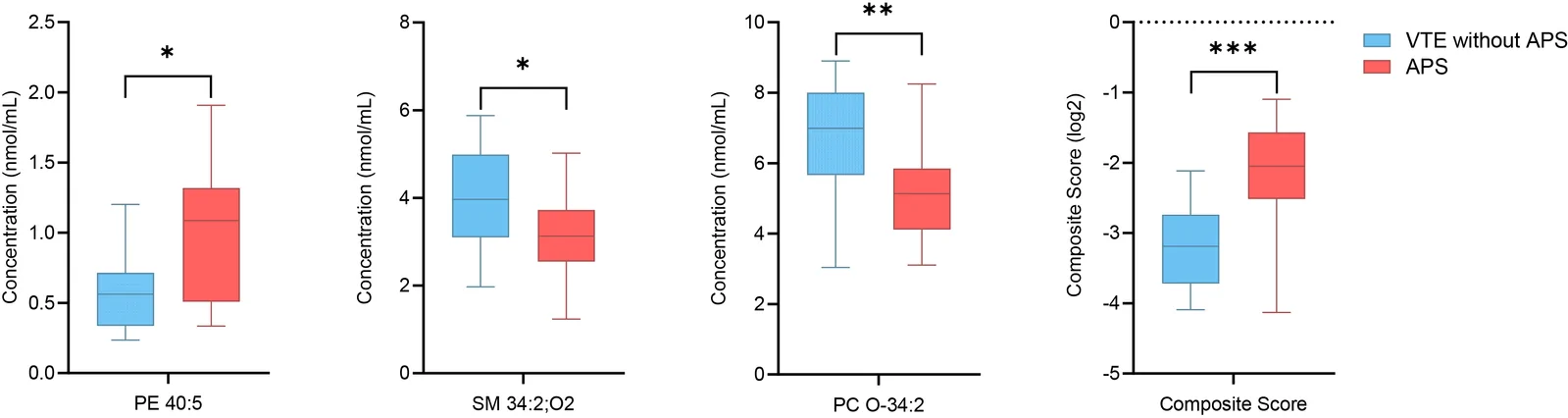
Plasma concentrations of LASSO-selected lipid species and 3-lipid composite score. Box plots display median with interquartile range; individual data points are shown as dots for PE 40:5, SM 43:2;O2, PC O-34:2 and the composite score S = log_2_(PE 40:5) − [log_2_(SM 43:2;O2) + log_2_(PC O-34:2)]/2 for APS patients and VTE patients without APS. Concentrations for PE 40:5 are analyzed by FIA-QQQ, SM 43:2;O2 and PC O-34:2 by FIA-FTMS. P-values were calculated using the Wilcoxon rank-sum test. ∗∗∗*P* < 0.001; ∗∗*P* < 0.01; ∗*P* < 0.05.

**Fig. 6:**
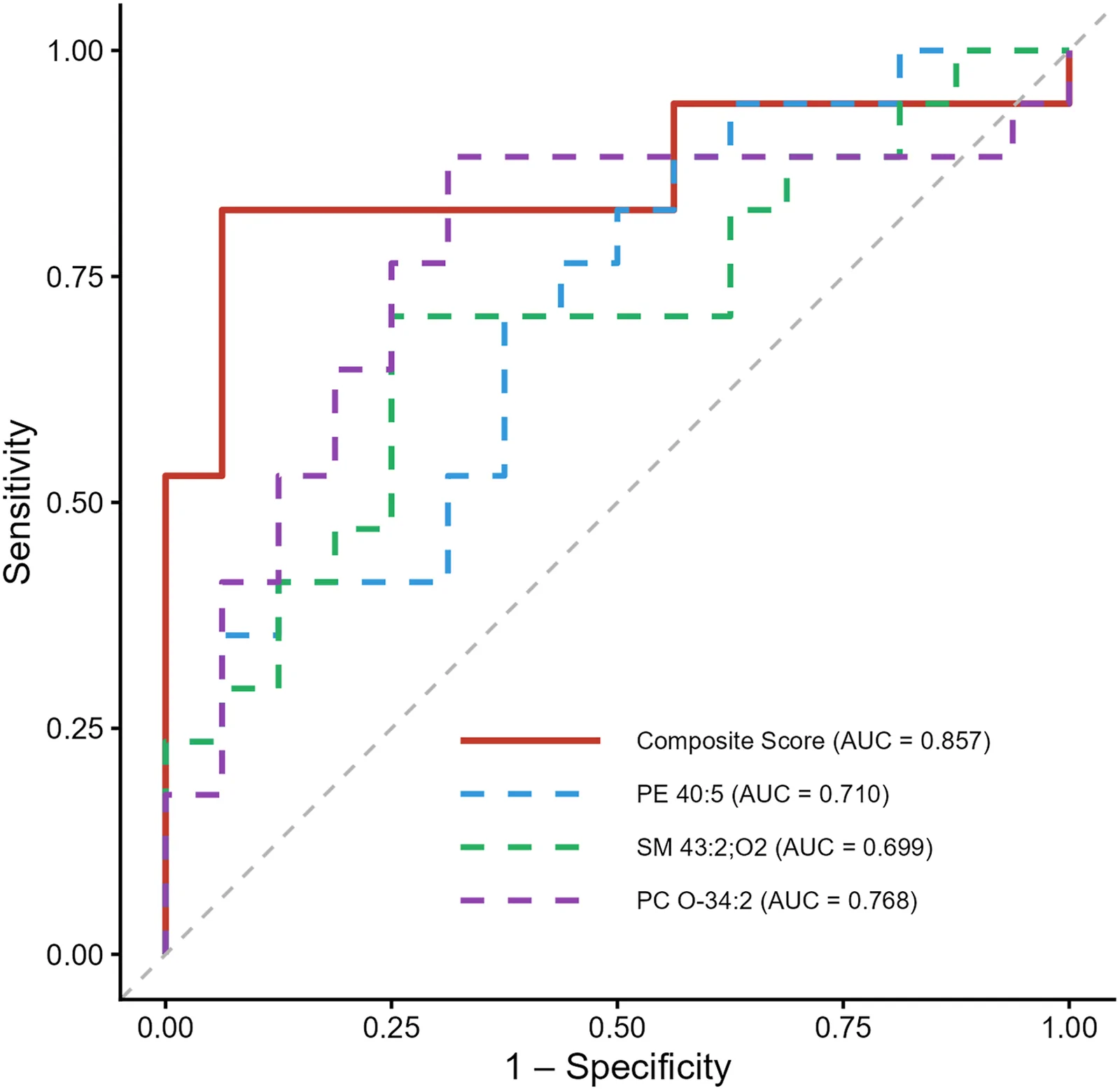
Receiver–operating characteristic (ROC) curves for discrimination of APS from VTE without APS. Individual curves are shown for PE 40:5, SM 43:2;O2, PC O-34:2; dashed lines) and for the composite lipid score derived from LASSO regression coefficients (solid red line). Area under the curve (AUC) values are indicated in the legend.

Despite the small number of patients in the current study, we explored whether lipid species could be applied to differentiate VTE patient groups with and without APS. To identify the most discriminating lipid panel whilst controlling for overfitting, LASSO logistic regression was applied to all species, identifying PE 40:5, SM 43:2;O2 and PC O-34:2 as the strongest discriminators (supplemental Table S2). PE 40:5 was 1.69-fold higher in APS (0.98 ± 0.50 vs. 0.58 ± 0.28 nmol/ml; AUC = 0.757, *P* = 0.011), while SM 43:2;O2 (3.12 ± 1.00 vs. 4.08 ± 1.19 nmol/ml; AUC = 0.732, *P* = 0.023) and PC O-34:2 (5.25 ± 1.29 vs. 6.68 ± 1.77 nmol/ml; AUC = 0.768, *P* = 0.008) were both reduced by approximately 21%–24% (Fig. 5). These three species were combined into a parameter-free composite score: S = log_2_(PE 40:5) − [log_2_(SM 43:2;O2) + log_2_(PC O-34:2)]/2 (Fig. 5). The composite score separated APS from VTE with an apparent AUC of 0.857 (Wilcoxon *P* = 2.5 × 10^-4^). Moreover, leave-one-out cross-validation of a logistic regression mapping S to group probability yielded an AUC of 0.824, confirming robustness. A permutation test (500 iterations, shuffled group labels, fixed score formula) yielded a permutation *P* < 0.002 (0 of 500 null AUCs exceeded the observed AUC), corroborating that the discriminative performance is unlikely to be due to chance (Fig. 6 and supplemental Table S2).

## Discussion

In the current study, we identified significant alterations in distinct lipid species in both VTE patients without APS and APS patients compared to healthy controls. A comprehensive analysis of the dataset revealed predominant alterations in ceramide concentrations across both thrombotic groups. It has been demonstrated that plasma concentrations of particular ceramide species are associated with cardiovascular and metabolic diseases, including obesity, insulin resistance and type 2 diabetes (reviewed in (24)). In the specific context of cardiovascular disease, the plasma concentrations of Cer 16:0, Cer 18:0 and Cer 24:1 have been identified as substantial predictors of a composite endpoint encompassing myocardial infarction, stroke, revascularisation and death from any cause, an association further strengthened by their ratios to Cer 24:0 (25). In the present study, Cer18:1;O2/16:0 and Cer18:1;O2/18:0 were elevated in both VTE patients without APS and APS patients relative to healthy controls, and an increase in the Cer 18:0/Cer 24:0 ratio was observed in both patient groups, suggesting a potential role for ceramide in venous thromboembolism. In support of a plausible mechanistic link, exogenous ceramide has been shown to induce the release of von Willebrand factor from endothelial cells in a dose-dependent manner (26), which may connect ceramides to factor VIII–dependent plasmatic haemostasis and thereby to VTE. Importantly, however, these ceramide alterations were not specific to APS and were equally present in VTE patients without APS, indicating that they reflect a general thrombotic state rather than a disease process unique to APS.

Beyond the ceramide alterations shared by both thrombotic groups, we identified a set of lipid species that specifically discriminated APS from VTE without APS. Exploratory unsupervised analyses (hierarchical clustering and principal component analysis) across all 224 quantified lipid species did not reveal a clear global separation between APS, VTE without APS, and healthy controls (data not shown). This indicates that APS is not characterized by a broad, lipidome-wide shift, but rather by alterations confined to a small number of specific lipid species. We therefore used targeted, regularized feature selection (LASSO), rather than global pattern recognition, to identify a discriminating lipid signature. Applying LASSO logistic regression to all measured species, we derived a three-lipid signature comprising PE 40:5, SM 43:2;O2 and PC O-34:2, which was combined into a parameter-free composite score. This score separated APS from VTE without APS with an apparent AUC of 0.857 (LOOCV AUC 0.824, permutation *P* < 0.002), demonstrating robust discriminative performance despite the limited cohort size. A study employing two-sample Mendelian randomization analysis has previously investigated associations between plasma lipid species and VTE risk, demonstrating a negative association of PC O-40:4 with VTE, while PC 42:6 and PC O-36:4 showed a positive association with pulmonary embolism (9). Of these species, only PC O-36:4 was quantified in our study; it trended higher in both VTE without APS and APS versus healthy volunteers (1.24- and 1.18-fold, respectively), directionally consistent with this association though not statistically significant. The same Mendelian randomization study also reported that higher fatty acid unsaturation (bis-allylic-to-double-bond ratio) was associated with increased risk of VTE, deep vein thrombosis, and pulmonary embolism. However, we found no significant differences in acyl chain unsaturation among PC, PC O, PE, or PI between our study groups. Rather than inferring causal associations from genetic instruments, we conducted direct lipidomic profiling of patient plasma with the specific aim of discriminating APS from other forms of VTE. A recent untargeted serum lipidomics study in obstetric APS reported a two-lipid diagnostic panel comprising PC 17:0/22:6 and ACar 17:3, achieving an AUC of 0.865 (27). However, the plausibility of this panel is questionable since ACar 17:3 is an odd-chain, polyunsaturated acylcarnitine that is exceptionally rare in human plasma, raising concerns about annotation accuracy for other lipid species as well. This reflects a broader challenge in untargeted lipidomics, where automated annotation algorithms have been shown to produce inconsistent results and may report lipid species unlikely to be present in biological samples (28).

Regarding the individual components of the three-lipid signature identified in our study, the elevation of PE 40:5 in APS plasma is consistent with a role for this species in cardiovascular inflammatory processes. Unfortunately, we could not confidently identify molecular lipid species for PE 40:5 in the product ion spectra acquired for selected samples. Literature data for NIST SRM 1950 plasma report more than 90% PE 18:0_22:4 and a minor fraction of PE 20:0_20:4 (29). In plasma lipidomic analyses of patients with stable and unstable coronary artery disease, PE 40:5, along with most other PE species, showed a positive association with stable coronary artery disease compared with controls (30). In a subsequent study examining lipoprotein-specific lipids in acute coronary syndrome, a twofold higher PE 40:5 concentration was detected in both the HDL and LDL fractions (31). A multi-omics investigation further identified a significant association between PE 40:5 and systolic blood pressure (32). Collectively, these data suggest that PE 40:5 is a marker of cardiovascular inflammatory activation, and its elevation in APS plasma may reflect the chronic thromboinflammatory state that characterizes this condition.

The reduction in PC O-34:2 in APS plasma indicates a disturbance in the ether phospholipid pool, as PC O-34:3, which was not selected for the lipid signature, was also found to be decreased. Product ion spectra revealed that PC O-34:2 is predominantly the alkyl ether (plasmanyl) species (>80% PC O-16:0/18:2). Alkyl-PC species (PC O) are metabolic precursors of platelet-activating factor (PAF), a potent pro-inflammatory and prothrombotic lipid mediator that activates multiple cell types including platelets, endothelial cells and monocytes (33). Reduced circulating PC O- species may reflect increased conversion to PAF under the inflammatory conditions that characterize APS, consistent with reports of elevated anti-PAF antibodies as independent risk factors for thrombosis (34) and reduced PAF acetylhydrolase (PAF-AH) activity in patients with antiphospholipid antibodies (35). These findings are further supported by our recent companion study, which demonstrated enhanced *in vivo* platelet activation in APS patients, as evidenced by elevated lysophospholipid/phospholipid ratios in the platelet lipidome (36). The lipidomics method applied in the present study cannot differentiate unsaturated alkyl and vinyl-ether bonds; therefore, PC O-34:3 may represent PC O-16:1/18:2 and/or plasmalogen PC P-16:0/18:2. Data for NIST SRM 1950 plasma did not report PC O-34:3, but PC P-16:0/18:2 (29). These data also showed that the concentration of PC O-34:2 was approximately 2.5 times higher than that of PC P-16:0/18:1, which aligns with our product ion spectra. Plasmalogens have been described as biomarkers in multiple diseases, partly due to their potential role as endogenous antioxidants (37, 38). In the context of APS, where oxidative stress and endothelial injury are well-recognized pathomechanisms, decreased levels of PC O-34:2 and PC O-34:3 may therefore reflect both substrate consumption for PAF synthesis and impaired antioxidant membrane defense.

The reduction of SM 43:2;O2 in APS plasma may reflect enhanced catabolism of sphingomyelin by secretory acid sphingomyelinase (ASMase), which has been shown to hydrolyze atherogenic lipoproteins (39). Notably, secreted ASMase activity is elevated in patients with acute coronary syndromes, where it associates with increased plasma ceramide levels (40). In this context, the co-occurrence in the present study of elevated ceramide species in both thrombotic groups and a specific further reduction of plasma SM 43:2;O2 in APS could be of interest. Since SM 43:2;O2 represents a minor SM species, we were not able to detect Cer 43:2;O2 as ASMase product. Furthermore, we could not identify an underlying sphingoid base in our product ion spectra. According to data from the LIPID MAPS consortium for NIST SRM 1950, there are five SM 43:2;O2 isomers, with SM 17:1;O2/26:1 (37% of the total) and SM 18:1;O2/25:1 (28% of the total) being the major isomers (41). Whether the selective decrease of SM 43:2;O2 reflects specific lipoprotein compositional changes or particular susceptibility of this species to sphingomyelinase activity remains unknown. Further studies including lipid profiling of lipoprotein subfractions and determination of ASMase activity in APS patient plasma are required to test this hypothesis.

The present study is limited by the small number of patients, which restricts the generalizability of the findings. Nevertheless, even within this cohort, the three-lipid composite score comprising PE 40:5, SM 43:2;O2 and PC O-34:2 achieved robust discrimination between APS and VTE, with an AUC of 0.857 confirmed by leave-one-out cross-validation and permutation testing. This suggests that the identified lipid signature captures biologically meaningful differences specific to APS. Validation in larger, independent cohorts is required to confirm these findings and to assess whether the composite score may be clinically useful for the stratification of VTE patients — in particular for identifying those with underlying APS who may benefit from targeted anticoagulation strategies.

In conclusion, using quantitative mass spectrometry-based lipidomics we have described specific alterations of distinct lipid species in plasma of VTE without APS and, to our knowledge for the first time, in APS patients. These findings support the need for future lipidomic studies in the context of VTE and APS to improve our understanding of the role of lipids in these diseases.

## Data availability

The data supporting this study are available in the article. Concentrations of the individual samples are available as supplemental Table S4. Further data are available from the corresponding author upon reasonable request.

## Supplemental data

This article contains supplemental data.

## Conflict of interest

The authors declare that they have no conflicts of interest with the contents of this article.
