# Supplementary material for: Plasma lipid alterations in thrombosis and antiphospholipid syndrome—A differentiating lipid plasma composite score
Source: J Lipid Res. 2026 Jul 31;67(9):101115. doi: 10.1016/j.jlr.2026.101115 (PMC13571436; doi:10.1016/j.jlr.2026.101115)
Supplement: Supplementary file 1 [file mmc5.pdf]

# Created by

Contents of Report https://lipidomicstandards.org, version v2.6.1


**Direct Infusion Workflow** 1
- Overall study design . . . 1
- Lipid extraction . . . 1
- Analytical platform . . . 1
- Quality control . . . 2
- Method qualification and validation . . . 2
- Reporting . . . 2

**Sample Descriptions** 2
- VTE-APS Study / Human / Plasma . . . 2

**Lipid Class Descriptions** 2
- 1) TG[M+NH4]+ / Lipid identification . . . 2
- 1) TG[M+NH4]+ / Lipid quantification . . . 3
- 2) CE[M+NH4]+ / Lipid identification . . . 3
- 2) CE[M+NH4]+ / Lipid quantification . . . 3
- 3) PC[M+HCOO]- / Lipid identification . . . 4
- 3) PC[M+HCOO]- / Lipid quantification . . . 4
- 4) SM[M+HCOO]- / Lipid identification . . . 4
- 4) SM[M+HCOO]- / Lipid quantification . . . 4
- 5) PC O[M+HCOO]- / Lipid identification . . . 5
- 5) PC O[M+HCOO]- / Lipid quantification . . . 5
- 6) FC[M+NH4]+ / Lipid identification . . . 5
- 6) FC[M+NH4]+ / Lipid quantification . . . 6
- 7) DG[M+NH4]+ / Lipid identification . . . 6
- 7) DG[M+NH4]+ / Lipid quantification . . . 6


# Direct Infusion Workflow

## Overall study design

| | | | |
|---|---|---|---|
| Title of the study | Plasma lipidomics VTE (FIA-FTMS) | | |
| Document creation date | 04/01/2026 | Principal investigator | Marcus Höring |
| Institution | Institute of Clinical Chemistry and Laboratory Medicine, University Hospital Regensburg | Corresponding Email | Marcus.Hoering@ukr.de |
| Is the workflow targeted or untargeted? | Untargeted | Clinical | Yes |

## Lipid extraction

| | | | |
|---|---|---|---|
| Extraction method | 2-phase system | pH adjustment | None |
| 2-phase system | Bligh&Dyer | Were internal standards added prior extraction? | Yes |

## Analytical platform

| | | | |
|---|---|---|---|
| Ionizaton additives | Ammonium formate | Detector | Mass spectrometer |
| MS type | Orbitrap | MS vendor | Thermo |
| Direct type | FIA | MS Level | $MS^1$, $MS^2$ |
| Mass resolution for detected ion at $MS^1$ | High resolution | Resolution at m/z 200 at $MS^1$ | 140.000 |
| Mass accuracy in ppm at $MS^1$ | 3 | Recording mode of raw data at $MS^1$ | Profile mode |
| Mass window for precursor ion isolation (in Da total isolation window) | 1 | Mass resolution for detected ion at $MS^2$ | High resolution |
| Resolution at m/z 200 at $MS^2$ | 140.000 | Mass accuracy in ppm at $MS^2$ | 3 |

| | | | |
|---|---|---|---|
| Recording mode of raw data at $MS^2$ | Profile mode | Was/Were additional dimension/techniques used | No |

## Quality control

| | | | |
|---|---|---|---|
| Blanks | Yes | Type of Blanks | Solvent blank, Internal standard blank |
| Quality control | Yes | Type of QC sample | Sample pool, Reference material |

## Method qualification and validation

| | | | |
|---|---|---|---|
| Method validation | Yes | Lipid recovery | Yes |
| Dynamic quantification range | No | Limit of quantitation (LOQ)/Limit of detection (LOD) | Yes |
| Precision | Yes | Accuracy | Yes |
| Guidelines followed | None | | |

## Reporting

| | | | |
|---|---|---|---|
| Are reported raw data uploaded into repository? | Available on request | Are metadata available? | Available on request |
| Raw data upload | Available on request | | |

# Sample Descriptions

## VTE-APS Study / Human / Plasma

| | | | |
|---|---|---|---|
| Storage and collection conditions | Available | Provided preanalytical information | Time to freeze, Storage time (month), Freeze-thaw cycles |
| Temperature handling original sample | Room temperature | Instant sample preparation | No |
| Time to freeze | between 60 and 120 min | Snap freezing in liquid N2 | No |
| Storage temperature | -80 °C | Storage time (month) | 30 |
| Freeze-thaw cycles | 1 | Additives | None |
| Were samples stored under inert gas? | No | Additional preservation methods | No |
| Biobank samples | No | | |

# Lipid Class Descriptions

## 1) TG[M+NH4]+ / Lipid identification

| | | | |
|---|---|---|---|
| Lipid class | TG | MS Level for identification | $MS^1$ |
| Identification level | Species level | $MS^1$ adduct | [M+NH4]+ |
| Isotope correction at $MS^1$ | No | $MS^1$ verified by standard | Yes |
| Background check at $MS^1$ | Yes | Did you presume assumptions for identification? | No |
| Check on: | Isomeric overlap, Isobaric overlap | Limit of detection | Signal threshold |
| Lipid Identification Software | ALEX123 | Data manipulation | Centroiding, Lock mass correction, Background subtraction |

| | |
|---|---|
| Nomenclature for intact lipid molecule | Yes |

## 1) TG[M+NH4]+ / Lipid quantification

| | | | |
|---|---|---|---|
| Quantitative | Yes | MS Level for quantification | $MS^1$ |
| Internal lipid standard(s) $MS^1$ | | | |

| Internal standard | Endogenous subclass |
|---|---|
| TG 51:0 | TG |
| TG 57:0 | TG |

| | | | |
|---|---|---|---|
| Type of quantification | Internal standard amount | Response correction | No |
| Type I isotope correction | Yes | Limit of quantification | Accuracy and coefficient of variation (< 20 %) |
| Normalization to reference | No | Lipid Quantification Software | Homemade |
| Batch correction | No | Further quantification remarks | Reported data quantified with TG 51:0 |

## 2) CE[M+NH4]+ / Lipid identification

| | | | |
|---|---|---|---|
| Lipid class | CE | MS Level for identification | $MS^1$ |
| Identification level | Species level | $MS^1$ adduct | [M+NH4]+ |
| Isotope correction at $MS^1$ | No | $MS^1$ verified by standard | Yes |
| Background check at $MS^1$ | Yes | Did you presume assumptions for identification? | No |
| Check on: | Isomeric overlap, Isobaric overlap, In-source fragmentation | Limit of detection | Signal threshold |
| Lipid Identification Software | ALEX123 | Data manipulation | Centroiding, Lock mass correction, Background subtraction |
| Nomenclature for intact lipid molecule | Yes | | |

## 2) CE[M+NH4]+ / Lipid quantification

| | | | |
|---|---|---|---|
| Quantitative | Yes | MS Level for quantification | $MS^1$ |
| Internal lipid standard(s) $MS^1$ | | | |

| Internal standard | Endogenous subclass |
|---|---|
| CE 17:0 | CE |
| CE 22:0 | CE |

| | | | |
|---|---|---|---|
| Type of quantification | Internal standard amount | Response correction | Response model |
| Type I isotope correction | Yes | Limit of quantification | Accuracy and coefficient of variation (< 20 %) |
| Normalization to reference | No | Lipid Quantification Software | Homemade |
| Batch correction | No | Further quantification remarks | Reported data quantified with CE 17:0 |

## 3) PC[M+HCOO]- / Lipid identification

| | | | |
|---|---|---|---|
| Lipid class | PC | MS Level for identification | $MS^1$ |
| Identification level | Species level | $MS^1$ adduct | [M+HCOO]- |
| Isotope correction at $MS^1$ | No | $MS^1$ verified by standard | Yes |
| Background check at $MS^1$ | Yes | Did you presume assumptions for identification? | Yes |
| Which assumptions were presumed? | Only even chain fatty acyl chains | Check on: | Isomeric overlap, Isobaric overlap |
| Limit of detection | Signal threshold | Lipid Identification Software | ALEX123 |
| Data manipulation | Centroiding, Lock mass correction, Background subtraction | Nomenclature for intact lipid molecule | Yes |
| Further identification remarks | Confirmation by positive ion mode and MS2 | | |

## 3) PC[M+HCOO]- / Lipid quantification

| | | | |
|---|---|---|---|
| Quantitative | Yes | MS Level for quantification | $MS^1$ |
| Internal lipid standard(s) $MS^1$ | | | |

| Internal standard | Endogenous subclass |
|---|---|
| PC 28:0 | PC |
| PC 44:0 | PC |

| | | | |
|---|---|---|---|
| Type of quantification | Internal standard amount | Response correction | No |
| Type I isotope correction | Yes | Limit of quantification | Accuracy and coefficient of variation (< 20 %) |
| Normalization to reference | No | Lipid Quantification Software | Homemade |
| Batch correction | No | Further quantification remarks | Reported data quantified with PC 28:0 |

## 4) SM[M+HCOO]- / Lipid identification

| | | | |
|---|---|---|---|
| Lipid class | SM | MS Level for identification | $MS^1$ |
| Identification level | Species level | $MS^1$ adduct | [M+HCOO]- |
| Isotope correction at $MS^1$ | No | $MS^1$ verified by standard | Yes |
| Background check at $MS^1$ | Yes | Did you presume assumptions for identification? | No |
| Check on: | Isomeric overlap, Isobaric overlap | Limit of detection | Signal threshold |
| Lipid Identification Software | ALEX123 | Data manipulation | Centroiding, Lock mass correction, Background subtraction |
| Nomenclature for intact lipid molecule | Yes | Further identification remarks | Confirmation by MS2 on a triple-quadrupole |

## 4) SM[M+HCOO]- / Lipid quantification

| | | | |
|---|---|---|---|
| Quantitative | Yes | MS Level for quantification | $MS^1$ |
| Internal lipid standard(s) $MS^1$ | | | |

| Internal standard | Endogenous subclass |
|---|---|
| SM 18:1;O2/12:0 | SM |
| SM 18:1;O2/18:1[D9] | SM |

| | | | |
|---|---|---|---|
| Type of quantification | Internal standard amount | Response correction | No |
| Type I isotope correction | Yes | Limit of quantification | Accuracy and coefficient of variation (< 20 %) |
| Normalization to reference | No | Lipid Quantification Software | Homemade |
| Batch correction | No | Further quantification remarks | Reported data quantified with SM 18:1;O2/18:1[D9] |

## 5) PC O[M+HCOO]- / Lipid identification

| | | | |
|---|---|---|---|
| Lipid class | PC O | MS Level for identification | $MS^1$ |
| Identification level | Species level | $MS^1$ adduct | [M+HCOO]- |
| Isotope correction at $MS^1$ | No | $MS^1$ verified by standard | Yes |
| Background check at $MS^1$ | Yes | Did you presume assumptions for identification? | Yes |
| Which assumptions were presumed? | Only even chain fatty acyl chains | Check on: | Isomeric overlap, Isobaric overlap |
| Limit of detection | Signal threshold | Lipid Identification Software | ALEX123 |
| Data manipulation | Centroiding, Lock mass correction, Background subtraction | Nomenclature for intact lipid molecule | Yes |
| Further identification remarks | Confirmation by positive ion mode and MS2 | | |

## 5) PC O[M+HCOO]- / Lipid quantification

| | | | |
|---|---|---|---|
| Quantitative | Yes | MS Level for quantification | $MS^1$ |

Internal lipid standard(s) $MS^1$

| Internal standard | Endogenous subclass |
|---|---|
| PC 28:0 | PC O- |
| PC 44:0 | PC O- |

| | | | |
|---|---|---|---|
| Type of quantification | Internal standard amount | Response correction | No |
| Type I isotope correction | Yes | Limit of quantification | Accuracy and coefficient of variation (< 20 %) |
| Normalization to reference | No | Lipid Quantification Software | Homemade |
| Batch correction | No | Further quantification remarks | Reported data quantified with PC 28:0 |

## 6) FC[M+NH4]+ / Lipid identification

| | | | |
|---|---|---|---|
| Lipid class | FC | MS Level for identification | $MS^2$ |
| Identification level | Species level | $MS^2$ adduct | [M+NH4]+ |

Fragments for identification

| Fragment name |
|---|
| Cholestadiene |

| | | | |
|---|---|---|---|
| Isotope correction at $MS^2$ | No | $MS^2$ verified by standard | Yes |
| Background check at $MS^2$ | Yes | Did you presume assumptions for identification? | No |

| | | | |
|---|---|---|---|
| Check on: | Isomeric overlap, Isobaric overlap, In-source fragmentation | Limit of detection | Signal threshold |
| Lipid Identification Software | ALEX123 | Data manipulation | Centroiding, Lock mass correction, Background subtraction |
| Nomenclature for intact lipid molecule | Yes | Nomenclature for fragment ions | N/A |
| Further identification remarks | multiplexing (MSX, n=2) of FC and the internal standard | | |

## 6) FC[M+NH4]+ / Lipid quantification

| | | | |
|---|---|---|---|
| Quantitative | Yes | MS Level for quantification | $MS^2$ |

Internal lipid standard(s) $MS^2$

| Internal standard | Fragment(s) | Endogenous subclass |
|---|---|---|
| FC[D7] | Cholestadiene[D7] | FC |

| | | | |
|---|---|---|---|
| Type of quantification | Internal standard amount | Response correction | No |
| Type I isotope correction | Yes | Limit of quantification | Accuracy and coefficient of variation (< 20 %) |
| Normalization to reference | No | Lipid Quantification Software | Homemade |
| Batch correction | No | Further quantification remarks | multiplexing (MSX, n=2) of FC and the internal standard FC[D7] |

## 7) DG[M+NH4]+ / Lipid identification

| | | | |
|---|---|---|---|
| Lipid class | DG | MS Level for identification | $MS^1$ |
| Identification level | Species level | $MS^1$ adduct | [M+NH4]+ |
| Isotope correction at $MS^1$ | No | $MS^1$ verified by standard | Yes |
| Background check at $MS^1$ | Yes | Did you presume assumptions for identification? | No |
| Check on: | Isomeric overlap, Isobaric overlap | Limit of detection | Signal threshold |
| Lipid Identification Software | ALEX123 | Data manipulation | Centroiding, Lock mass correction, Background subtraction |
| Nomenclature for intact lipid molecule | Yes | | |

## 7) DG[M+NH4]+ / Lipid quantification

| | | | |
|---|---|---|---|
| Quantitative | Yes | MS Level for quantification | $MS^1$ |

Internal lipid standard(s) $MS^1$

| Internal standard | Endogenous subclass |
|---|---|
| DG 28:0 | DG |
| DG 40:0 | DG |

| | | | |
|---|---|---|---|
| Type of quantification | Internal standard amount | Response correction | No |
| Type I isotope correction | Yes | Limit of quantification | Accuracy and coefficient of variation (< 20 %) |
| Normalization to reference | No | Lipid Quantification Software | Homemade |
| Batch correction | No | Further quantification remarks | Reported data quantified with DG 28:0 |
