# Supplementary material for: Plasma lipid alterations in thrombosis and antiphospholipid syndrome—A differentiating lipid plasma composite score
Source: J Lipid Res. 2026 Jul 31;67(9):101115. doi: 10.1016/j.jlr.2026.101115 (PMC13571436; doi:10.1016/j.jlr.2026.101115)
Supplement: Supplementary file 2 [file mmc6.pdf]

Created by

Direct Infusion Workflow 1
Overall study design . . . 1
Lipid extraction . . . 1
Analytical platform . . . 1
Quality control . . . 2
Method qualification and validation . . . 2
Reporting . . . 2

Sample Descriptions 2
VTE-APS Study / Human / Plasma . . . 2

Lipid Class Descriptions 2
1) PE[M+H]+ / Lipid identification . . . 2
1) PE[M+H]+ / Lipid quantification . . . 3
2) LPE[M+H]+ / Lipid identification . . . 3
2) LPE[M+H]+ / Lipid quantification . . . 3
3) LPC[M+H]+ / Lipid identification . . . 4
3) LPC[M+H]+ / Lipid quantification . . . 4
4) PI[M+NH4]+ / Lipid identification . . . 4
4) PI[M+NH4]+ / Lipid quantification . . . 5
5) Cer[M+H]+ / Lipid identification . . . 5
5) Cer[M+H]+ / Lipid quantification . . . 5
6) HexCer[M+H]+ / Lipid identification . . . 6
6) HexCer[M+H]+ / Lipid quantification . . . 6
7) PE P[M+H]+ / Lipid identification . . . 6
7) PE P[M+H]+ / Lipid quantification . . . 7

Direct Infusion Workflow

Overall study design

Title of the study	Plasma lipidomics VTE (FIA-QQQ)		
Document creation date	06/29/2026	Principal investigator	Gerhard Liebisch
Institution	Institute of Clinical Chemistry and Laboratory Medicine, University Hospital Regensburg	Corresponding Email	gerhard.liebisch@ukr.de
Is the workflow targeted or untargeted?	Targeted	Clinical	Yes

Lipid extraction

Extraction method	2-phase system	pH adjustment	None
2-phase system	Bligh&Dyer	Were internal standards added prior extraction?	Yes

Analytical platform

Ionizaton additives	Ammonium acetate	Detector	Mass spectrometer
MS type	QQQ	MS vendor	Waters
Direct type	FIA	MS Level	MS^2
Mass window for precursor ion isolation (in Da total isolation window)	0.8	Mass resolution for detected ion at MS^2	Low resolution
Resolution at MS^2	Unit	Recording mode of raw data at MS^2	Profile mode
Was/Were additional dimension/techniques used	No		

Quality control

Blanks	Yes	Type of Blanks	Solvent blank, Internal standard blank
Quality control	Yes	Type of QC sample	Sample pool, Reference material

Method qualification and validation

Method validation	Yes	Lipid recovery	Yes
Dynamic quantification range	Yes	Limit of quantitation (LOQ)/Limit of detection (LOD)	Yes
Precision	Yes	Accuracy	Yes
Guidelines followed	None		

Reporting

Are reported raw data uploaded into repository?	Available on request	Are metadata available?	Available on request
Raw data upload	Available on request		

Sample Descriptions

VTE-APS Study / Human / Plasma

Storage and collection conditions	Available	Provided preanalytical information	Time to freeze, Storage time (month), Freeze-thaw cycles
Temperature handling original sample	Room temperature	Instant sample preparation	No
Time to freeze	between 60 and 120 min	Snap freezing in liquid N2	No
Storage temperature	-80 °C	Storage time (month)	30
Freeze-thaw cycles	1	Additives	None
Were samples stored under inert gas?	No	Additional preservation methods	No
Biobank samples	No		

Lipid Class Descriptions

1) PE[M+H]+ / Lipid identification

Lipid class	PE	MS Level for identification	MS^2
Identification level	Species level	MS^2 adduct	[M+H]+
Fragments for identification			

Fragment name
-HG(PE,141)

Isotope correction at MS^2	Type 2	MS^2 verified by standard	Yes
Background check at MS^2	Yes	Did you presume assumptions for identification?	Yes
Which assumptions were presumed?	Presence of acyl-bond for all species	Check on:	Isomeric overlap, Isobaric overlap
Limit of detection	Signal threshold	Lipid Identification Software	Homemade

Data manipulation	Smoothing, Centroiding, Background subtraction	Nomenclature for intact lipid molecule	Yes
Nomenclature for fragment ions	N/A		

1) PE[M+H]+ / Lipid quantification

Quantitative	Yes	MS Level for quantification	MS^2

Internal lipid standard(s) MS^2

Internal standard	Fragment(s)	Endogenous subclass
PE 14:0/14:0, PE 20:0/20:0 (di-phytanoyl)	-HG(PE,141)	all PE species

Type of quantification	Internal standard amount	Response correction	No
Type I isotope correction	Yes	Limit of quantification	Signal threshold
Normalization to reference	Yes	Lipid Quantification Software	Homemade
Batch correction	No	Further quantification remarks	PE 20:0/20:0 was preferred for quantification, the second standard was used for sample internal QC-check.

2) LPE[M+H]+ / Lipid identification

Lipid class	LPE	MS Level for identification	MS^2
Identification level	Species level	MS^2 adduct	[M+H]+

Fragments for identification

Fragment name
-HG(PE,141)

Isotope correction at MS^2	Type 2	MS^2 verified by standard	Yes
Background check at MS^2	Yes	Did you presume assumptions for identification?	Yes
Which assumptions were presumed?	Presence of acyl-bond for all species	Check on:	Isomeric overlap, Isobaric overlap
Limit of detection	Signal threshold	Lipid Identification Software	Homemade
Data manipulation	Smoothing, Centroiding, Background subtraction	Nomenclature for intact lipid molecule	Yes
Nomenclature for fragment ions	N/A		

2) LPE[M+H]+ / Lipid quantification

Quantitative	Yes	MS Level for quantification	MS^2

Internal lipid standard(s) MS^2

Internal standard	Fragment(s)	Endogenous subclass
LPE 13:0, LPE 18:1[D7]	-HG(PE,141)	all LPE species

Type of quantification	Internal standard amount	Response correction	No
Type I isotope correction	Yes	Limit of quantification	Signal threshold
Normalization to reference	Yes	Lipid Quantification Software	Homemade
Batch correction	No	Further quantification remarks	LPE 18:1[D7]was preferred for quantification, the second standard was used for sample internal QC-check.

3) LPC[M+H]+ / Lipid identification

Lipid class	LPC	MS Level for identification	MS^2
Identification level	Species level	MS^2 adduct	[M+H]+

Fragments for identification

Fragment name
HG(PC,184)

Isotope correction at MS^2	Type 2	MS^2 verified by standard	Yes
Background check at MS^2	Yes	Did you presume assumptions for identification?	Yes
Which assumptions were presumed?	Presence of acyl-bond for all species	Check on:	Isomeric overlap, Isobaric overlap
Limit of detection	Signal threshold	Lipid Identification Software	Homemade
Data manipulation	Smoothing, Centroiding, Background subtraction	Nomenclature for intact lipid molecule	Yes
Nomenclature for fragment ions	N/A		

3) LPC[M+H]+ / Lipid quantification

Quantitative	Yes	MS Level for quantification	MS^2

Internal lipid standard(s) MS^2

Internal standard	Fragment(s)	Endogenous subclass
LPC 13:0, LPC 19:0,	HG(PC,184)	all LPC species

Type of quantification	Internal standard amount	Response correction	No
Type I isotope correction	Yes	Limit of quantification	Signal threshold
Normalization to reference	Yes	Lipid Quantification Software	Homemade
Batch correction	No	Further quantification remarks	LPC 13:0 was preferred for quantification, the second standard was used for sample internal QC-check.

4) PI[M+NH4]+ / Lipid identification

Lipid class	PI	MS Level for identification	MS^2
Identification level	Species level	MS^2 adduct	[M+NH4]+

Fragments for identification

Fragment name
-HG(PI,277)

Isotope correction at MS^2	Type 2	MS^2 verified by standard	Yes
Background check at MS^2	Yes	Did you presume assumptions for identification?	Yes
Which assumptions were presumed?	Presence of acyl-bond for all species	Check on:	Isomeric overlap, Isobaric overlap
Limit of detection	Signal threshold	Lipid Identification Software	Homemade
Data manipulation	Smoothing, Centroiding, Background subtraction	Nomenclature for intact lipid molecule	Yes

Nomenclature for fragment ions	N/A		

4) PI[M+NH4]+ / Lipid quantification

Quantitative	Yes	MS Level for quantification	MS^2

Internal lipid standard(s) MS^2

Internal standard	Fragment(s)	Endogenous subclass
PI 15:0/18:1[D7]	-HG(PI,277)	all PI species

Type of quantification	Internal standard amount	Response correction	No
Type I isotope correction	Yes	Limit of quantification	Signal threshold
Normalization to reference	Yes	Lipid Quantification Software	Homemade
Batch correction	No		

5) Cer[M+H]+ / Lipid identification

Lipid class	Cer	MS Level for identification	MS^2
Identification level	Species level	MS^2 adduct	[M+H]+

Fragments for identification

Fragment name
LCB 18:1;O2(-H3O2)

Isotope correction at MS^2	Type 2	MS^2 verified by standard	Yes
Background check at MS^2	Yes	Did you presume assumptions for identification?	No
Check on:	Isomeric overlap, Isobaric overlap, In-source fragmentation	Limit of detection	Signal threshold
Lipid Identification Software	Homemade	Data manipulation	Smoothing, Centroiding, Background subtraction
Nomenclature for intact lipid molecule	Yes	Nomenclature for fragment ions	N/A
Further identification remarks	Both [M+H]+ and [M+H-H2O]+ were acquired.		

5) Cer[M+H]+ / Lipid quantification

Quantitative	Yes	MS Level for quantification	MS^2

Internal lipid standard(s) MS^2

Internal standard	Fragment(s)	Endogenous subclass
Cer 18:1;O2/17:0, Cer 18:1;O2[D7]/17:0	LCB 18:1;O2(-H3O2), LCB 18:1;O2[D7](-H3O2)	all Cer species

Type of quantification	Internal standard amount	Response correction	No
Type I isotope correction	Yes	Limit of quantification	Signal threshold
Normalization to reference	Yes	Lipid Quantification Software	Homemade
Batch correction	No	Further quantification remarks	Cer 18:1;O2[D7]/17:0 was preferred for quantification, the second standard was used for sample internal QC-check. Sum of [M+H]+ and [M+H-H2O]+ was used for quantification.

6) HexCer[M+H]+ / Lipid identification

Lipid class	HexCer	MS Level for identification	MS^2
Identification level	Species level	MS^2 adduct	[M+H]+

Fragments for identification

Fragment name
LCB 18:1;O2(-H3O2)

Isotope correction at MS^2	Type 2	MS^2 verified by standard	Yes
Background check at MS^2	Yes	Did you presume assumptions for identification?	No
Check on:	Isomeric overlap, Isobaric overlap, In-source fragmentation	Limit of detection	Signal threshold
Lipid Identification Software	Homemade	Data manipulation	Smoothing, Centroiding, Background subtraction
Nomenclature for intact lipid molecule	Yes	Nomenclature for fragment ions	N/A
Further identification remarks	Both [M+H]+ and [M+H-H2O]+ were aquired.		

6) HexCer[M+H]+ / Lipid quantification

Quantitative	Yes	MS Level for quantification	MS^2

Internal lipid standard(s) MS^2

Internal standard	Fragment(s)	Endogenous subclass
GlcCer 18:1;O2/17:0, GlcCer 18:1;O2[D5]/18:0	LCB 18:1;O2(-H3O2), LCB 18:1;O2[D5](-H3O2)	all HexCer species

Type of quantification	Internal standard amount	Response correction	No
Type I isotope correction	Yes	Limit of quantification	Signal threshold
Normalization to reference	Yes	Lipid Quantification Software	Homemade
Batch correction	No	Further quantification remarks	GlcCer 18:1;O2[D5]/18:0 was preferred for quantification, the second standard was used for sample internal QC-check. Sum of [M+H]+ and [M+H-H2O]+ was used for quantification.

7) PE P[M+H]+ / Lipid identification

Lipid class	PE P	MS Level for identification	MS^2
Identification level	Species level	MS^2 adduct	[M+H]+

Fragments for identification

Fragment name
Plasmalogen specific fragment

Isotope correction at MS^2	Type 2	MS^2 verified by standard	Yes
Background check at MS^2	Yes	Did you presume assumptions for identification?	Yes

Which assumptions were presumed?	Presence of acyl-bond for all species	Check on:	Isomeric overlap, Isobaric overlap
Limit of detection	Signal threshold	Lipid Identification Software	Homemade
Data manipulation	Smoothing, Centroiding, Background subtraction	Nomenclature for intact lipid molecule	Yes
Nomenclature for fragment ions	N/A		

7) PE P[M+H]+ / Lipid quantification

Quantitative	Yes	MS Level for quantification	MS^2

Internal lipid standard(s) MS^2

Internal standard	Fragment(s)	Endogenous subclass
PE 14:0/14:0, PE 20:0/20:0 (di-phytanoyl)	Plasmalogen specific fragment	all PE P species

Type of quantification	Internal standard amount	Response correction	No
Type I isotope correction	Yes	Limit of quantification	Signal threshold
Normalization to reference	Yes	Lipid Quantification Software	Homemade
Batch correction	No	Further quantification remarks	PE 20:0/20:0 (di-phytanoyl) was preferred for quantification, the second standard was used for sample internal QC-check.
